# The Intensity of Formal Child-Care Attendance Decreases the Shared Environment Contribution to School Readiness: A Twin Study

**DOI:** 10.1007/s10578-022-01440-6

**Published:** 2022-10-21

**Authors:** Eloi Gagnon, Michel Boivin, Catherine Mimeau, Bei Feng, Genevieve Morneau-Vaillancourt, Sophie Aubé, Mara Brendgen, Frank Vitaro, Ginette Dionne

**Affiliations:** 1 Department of Psychology, Laval University, 2325 Allée des Bibliothèques, Québec, QC G1V 0A6 Canada; 2 Department of Psychology, Quebec University in Montreal, Montreal, QC Canada; 3 School of Psychoeducation, University of Montreal, Montreal, QC Canada

**Keywords:** Child-care, School readiness, GxE interaction, Twin study

## Abstract

The purpose of this study was to explore if child-care intensity (hours/weeks) and age of onset could moderate genetic and environmental contributions to school readiness. A sample of 648 (85% Whites; 50% Females) pairs of twins was used to compute a GxE, CxE and ExE interaction analyses. The moderation model showed that shared environment explains 48% of individual differences in school readiness for children not attending formal child-care, and decreased gradually to a mere 3% for children attending formal child-care full time, e.g., 40 h per week. Age of onset exerted no moderation effect. The results support the hypothesis that child-care acts as a normalizing environment, possibly buffering negative effects from low-quality home environments on school readiness.

**Supplementary Information:**

The online version contains supplementary material available at 10.1007/s10578-022-01440-6.

## Study Rationale

Like other developed countries, the majority (53%) of Canadian children under 5 years of age attend child-care [1, 2]. As child-care has become more common in the recent decades due to an increasing number of mothers entering the workforce, researchers, policy-makers and educators have become increasingly interested in documenting its effects on children’s development. Cohort studies and randomized control studies have shown that good quality child-care enhances preschool and school-age cognitive abilities, especially for children from low socioeconomic status (SES) households [3]. In particular, good child-care can compensate, at least partially, for being raised in low SES households [4]. Gene-sensitive study could help determining the genetic and environmental pathways through which formal child-care attendance may play a role in school readiness. A gene-sensitive study on American twins noticed that child-care attendance at four years of age tended to decrease shared environmental influence on academic achievement [5]. This result is in line with the idea that child-care contributes the most to children from low SES family backgrounds by decreasing negative family-level influence. However, it is unclear if this effect can be replicated in a different set of twins and if different patterns will emerge by exploring continuous measures such as age of onset and the intensity of child-care attendance. Therefore, this study aimed to explore if child-care attendance (from 6 to 50 months of age) can moderate genetic and environmental contributions to school readiness at 60 months in the Quebec Newborn Twin Study (QNTS), a prospective, population-based and genetically informative sample of 662 pairs of twins.

Child-care can be defined as services provided by someone other than the parents to care for and educate children in their absence. This includes formal (center-based) and informal (e.g., grandparent custody, nanny) care. Early studies focused on developmental differences between children attending and not attending child-care [6]. Later work recognized that child-care experience is not homogenous and that the characteristics of the experience matter [7]. In particular, two dimensions of child-care have emerged as long-term predictors of child development: quality and quantity [8]. Quality of child-care refers to its characteristics such as caregivers’ training, child-staff ratio and child-staff relationship [8]. Quantity refers to the amount of time experienced in child-care by the child, including age of onset and number of hours per week [9]. Both quality and quantity play a significant role in children’s child-care experience.

The role of child-care regarding school readiness has been extensively studied [10]. Good quality child-care has repeatedly been associated with better child outcomes, including school readiness and language development [8], higher academic grades, and admission to more selective colleges [11]. Also, child-care quality appears to interact with its quantity: under low quality, high quantity yields no or even negative outcomes, whereas under high quality, high quantity can yield positive outcomes [12]. Indeed, child-care attendance, high number of hours of attendance per week, and early age of onset have been all positively associated with preschool receptive vocabulary and semantic fluency, especially when child-care quality is high [13–15].

In Quebec, child-care is subsidized and organized into centers with specific educational programs. However, access to these programs is not universal, mainly because of the lack of places, but also because some parents choose other means of child-care. A provincial survey conducted in 2003 (three years after the QNTS cohort finished child-care) showed that, formal center-based child-care is by far the most widely used type of child-care in Quebec (70%) [16]. They were also found to provide the best quality of child-care [16]. Quality differed by type of child-care services: family type child-care settings were rated on average as acceptable, whereas public center-based child-care settings were typically rated as "good," and in few cases, "excellent". Hence, in population-based studies, type of child-care has been repeatedly used as a proxy for quality [4, 17, 18]. These former studies reported significant more positive outcomes for formal versus informal child-care with respect to cognitive school readiness, receptive vocabulary, number knowledge, and reading performance at ages 6 and 7 years, as well as provincial standardized exams at 12 years of age. Focusing solely on the widely available formal child-care in the Quebec population may yield to a less heterogeneous construct in terms of quality.

### The Twin Model to Assess the Contribution of Genetic and Environmental Factors

Genetically informed study designs using samples of twins have contributed to this research area by exploring genetic etiology of school readiness. The twin model uses the natural occurrence of MZ twins, who share 100% of their genetic background, and dizygotic (DZ) twins, who share on average 50% of their genetic background to estimate genetic and environmental contributions to individual differences. Covariation among twins from the same family are calculated for a given trait. The extent to which MZ twins are more similar than DZ twins indicates a contribution of genes (A), heritability, to the variance of a given phenotype. The remaining variance can be explained by shared environment (C) that yields similarities between twins, and non-shared environment (E) that yields differences between twins regardless of zygosity.

School readiness is one of the rare constructs in behavioral genetic with a predominant shared environment contribution [19]. In the preschool years, individual differences in school readiness have been shown to be accounted for mostly by shared environmental factors (53%) and to a lesser extent by genetic (32%) and unique environment (15%) factors [20]. Math skills at four years of age display the same pattern where shared environment is predominant compared to genes and unique environment [21, 22]. Reading skills follow a similar pattern in which shared environment factors contribute to most of the variance (54%) seconded by unique environment (21%) and additive genetic factor (18%) [22].

The partition of variance into genetic and environmental components assumes additivity of their contributions. However, it is possible that their contributions do not simply add up and vary as a function of contexts. Indeed, the contributions of A, C and E could vary as a function of a specific environmental exposure. For example, a significant Gene-Environment (GxE) interaction suggests that the effect of the environment depends on genes, or conversely that the effect of genes depends on the environment [23–25]. It applies to patterns in which genetically gifted individuals thrive in their school readiness despite a low-quality environment that negatively affect others, or in which individuals with inherited learning difficulties continue to struggle despite the optimal support that benefits others.

Surprisingly, GxE interaction in the context of child-care has been sparsely studied. We found only two studies that tested GxE interaction in the context of child-care. Both examined the same set of 600 twins from the United States who attended or not child-care at age four, but did not examine continuous moderators [5, 26]. The first study tested the hypothesis of a GxE interaction in externalizing behavior. The authors found that attending child-care at age four was associated with lower shared environment contribution and higher genetic contribution to externalizing behavior at age five. Among children who did not attend child-care, externalizing at age 5 was predominantly due to environmental influences (52% shared environment, 34% non-shared environment) rather than genetic influences (13%), whereas among children who had attended preschool at 4, genetic influence on externalizing was higher (67%), and shared environmental influences were smaller (0%) [26].

The second study, particularly relevant for the present work, investigated GxE interaction with child-care and reading and math scores at age four and five. The author found that child-care presence at four was associated with a decrease in shared environmental contribution to math and reading scores but not genetic and unique environmental contributions. Among children who did not attend preschool at age 4, shared environmental influences accounted for 72% of the variance in math scores and 73% of the variance in reading scores at age 5. These contributions were significantly smaller among children who attended preschool at age 4: shared environmental influences accounted for 47% of the variance in math scores, as well as for 43% of the variance in reading scores at age five [22].

Overall, the results from the two studies above suggest that attending child-care may diminish the role of shared environmental in academic achievement at five, but these results suffer from several limitations that we tried to address here. Tucker-Drob compared the genetic and environmental contributions to school abilities of children who did and did not attend child-care at four years of age, and thus only examined the binary role of child-care presence without evaluating earlier enrollment. In the present study, we address these limitations and expand on the aforementioned studies in three important ways. First, we evaluate multiple aspects of child-care attendance, including child-care intensity and age of onset, to gain a broader and more complete overview of child-care experience and to better understand how these characteristics of child-care experience may translate into potential developmental effects on school readiness. Second, whereas previous studies evaluated child-care attendance at a single time point, we use multiple follow-up assessments of child-care attendance between 6 and 50 months old, providing a more detailed portrait of child-care attendance during early childhood. Third, in contrast to previous studies which used a binary measure of child-care (i.e., yes or no), we provide a finer measure for child-care attendance by including continuous measures of child-care attendance. Simulations have shown to be a good strategy to adopt continuous moderators whenever available, thereby increasing the power to detect a continuous moderation [27]. Fourth, the inclusion of measures of intensity allows to uncover more precisely which aspect of child-care moderates the variance of school readiness.

### Objectives of the Present Study

Twin studies show that shared environmental factors significantly account for school readiness, suggesting that adverse familial environment may play a central role in explaining differences between children in terms of cognitive abilities at school entry [20]. Yet, preliminary evidence suggests that formal child-care attendance in early childhood may play a substantial role in decreasing the contribution from family-wide environmental factors to school readiness. Using data from the QNTS, a prospective longitudinal and population-based study, the present confirmatory study explored whether the quantity of child-care (age of onset, number of hours) moderate genetic and environmental contributions to school readiness. Based on previous work [5], we hypothesize that attending child-care will be associated with lower shared environment contribution to school readiness.

## Method

### Participants

The present study used data from the QNTS, a population-based longitudinal study which initially recruited 662 twin pairs at birth between April 1995 and December 1998 in the Greater Montreal Area, Canada [28]. Cognitive and environmental data including child-care attendance have been collected quasi-yearly from the twins and their parents from 6 to 60 months of age and beyond. The initial QNTS sample was representative of the Quebec population in terms of family educational attainment and ethnicity [29]. At the first wave, the average household income was approximately CAN $54,000, which is slightly higher than the average income of families in the same region at that time [29]. Consent was obtained from the twins’ parents prior to each data collection. Analyses were performed on a subsample of 648 twin pairs (245 MZ pairs and 403 DZ pairs) for whom we had data on child-care attendance at one or more time points when twins were 6 months (*M* = *6.3, SD* = *0.73*), 20 months (*M* = *19.6, SD* = *0.82*), 32 months (*M* = *31.9, SD* = *0.99*), and 50 months old (*M* = *50.2, SD* = *1.9*). We used all available data and managed missing data with full information maximum likelihood to maximize power.

### Measures

#### Child-Care Attendance

At the child’s ages 6, 20, 32 and 50 months, mothers reported on child-care attendance and the weekly number of hours their child spent in various child-care settings. We derived the following two formal child-care quantity variables from participants with child-care attendance at all four-time points (N = 770): (1) intensity and (2) age of onset. We operationalized intensity similarly to other studies on quantity of care [30]. Formal child-care intensity was the average number of hours per week of child-care attendance across the four-time points (*skewness* = 1.39; *kurtosis* = 1.16). Age of onset was the age of the child when entering a formal school like setting for the first time (*skewness* = − 0.60; *kurtosis* = − 0.91). Children who never attended child-care, but attended kindergarten were given the age at which they entered kindergarten (*M* = *64.49, SD* = *3.16*) months. Indeed, although kindergarten is not mandatory, 98.1% of eligible children were enrolled in 2002–2003 [31], time at which participants enrolled in kindergarten. The participants with child-care attendance at all four-time points (N = 770) did not differ in terms of school readiness and child-care attendance at all waves of assessment compared to the original sample. As twin concordance was very high for child-care attendance (*r* = 0.99), we computed an interfamily score by averaging the child-care attendance means for both twins. Thus, child-care attendance was used as a family-wide environmental moderator in GxE testing.

#### School Readiness

Twins were administered the Lollipop test, a widely used measure of school readiness, in a laboratory setting at 60 months, the summer before school entry [32, 33]. This measure has been shown to successfully predict early school achievement [20, 34]. Twin pairs were tested by two different testers, blind to the status of the twins (MZ versus DZ), in two different rooms, to avoid inflated twin similarity. The test was administered in French (85%) or English (15%), depending on the predominant language of the child. We included school readiness data from 908 (384 monozygotic, 524 dizygotic) twins. The Lollipop test consists of 52 items divided into four subtests each assessing a component of cognitive school readiness: (a) Identification of Colors and Shapes; (b) Picture Description and Spatial Recognition; (c) Identification of Numbers and Counting; and (d) Identification of Letters and Writing. An overall score can be obtained by combining the scores of the four subtests. The scores for subtests 1 to 3 range from 0 to 17, scores for subtest 4 range from 0 to 18, and overall scores range from 0 to 69. The overall score was used in the present study. Cronbach alpha for this total score was $\alpha =.73$. The skewness was − 0.20 and kurtosis was − 0.61.

#### Control Variables

Because the parents’ choice to send their children to child-care is not random, we controlled for several selection factors susceptible to affect child-care attendance in our GxE model. Selection factors are variables predicting the parents’ choice of child-care intensity. We examined 14 selection factors that have previously been associated with child-care attendance in a demographically and geographically similar sample [17]. The following selection factors were assessed by the mother when her twins were five months old, unless otherwise specified: (a) *siblings*, that is, the average of the number of siblings from 0 to 18 years of age living in the child’s household at 6 and 50 months; (b) *family income* assessed on an 11-item scale ranging from no income to above $80,000 per year at the first four-time points. Income at the four-time points were averaged. (c) *marital status*, that is, whether the family unit consisted of two biological parents or not; (d) *health at birth* (i.e., “Compared to other babies in general, how is your child’s health at birth?”) assessed on a five-point scale ranging from poor (1) to excellent (5); (e) *ethnicity*, that is, whether or not the child is White (proportion of White = 85%); (f) *pregnancy smoking*, that is, the average number of cigarettes smoked per day throughout the whole pregnancy; (g) *breastfeeding*, that is, whether or not the mother had breastfed the child; (h) birth weight measured in grams from medical birth record; (i) *maternal* *age* at the twins’ birth measured in years; (j) *gestational age* in months at the first wave of assessment from medical birth record; (k) *sex*; (l) *family functioning* assessed with a subset of the family assessment device which comprises 12 items reflecting the quality of family relationships, such as problem solving, communication, roles, affective responsiveness, and behavioral control [35], with scores ranging from 0 to 30, a high value indicating a dysfunctional family environment; (m) *maternal education*, that is, the number of years of education of the mother reported at 60 months; (n) *paternal education*, that is, the number of years of education of the father reported by the father at 60 months.

### Statistical Analyses

All summary statistics and phenotypical analysis were performed in the open-source statistical computing environment *R* [36]. Structural equation modeling applied to the twin model provides an efficient way to estimate genetic and environmental contributions to variance and covariance in a phenotype [37]. We used Structural Equation and Twin Modeling in *Mplus* version 8.1 to fit structural equation models to the phenotypic covariance structure between twins.

**Fig. 1:**
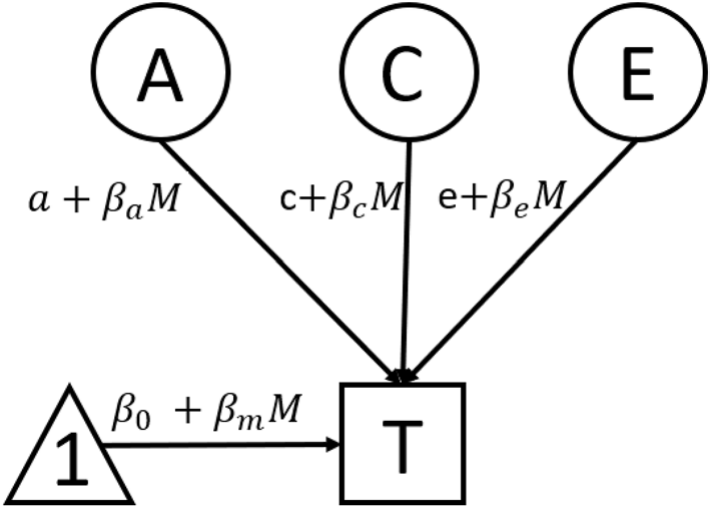
Path diagram of the GxE model for one twin. *Note* Only one twin is shown for simplicity. $\mathrm{M}$ = moderator; $\mathrm{a}$, $\mathrm{c}$, $\mathrm{e}$ = unmoderated genetic, shared environment, and unique environment components, respectively; ${\upbeta }_{\mathrm{a}}$, ${\upbeta }_{\mathrm{c}}$, ${\upbeta }_{\mathrm{e}}$ = moderator effect on the $\mathrm{a}$, $\mathrm{c}$,and $\mathrm{e}$, respectively; ${\upbeta }_{0}$ = mean effect intercept; ${\upbeta }_{\mathrm{m}}$ = mean linear effect of the moderator on the trait

We first estimated a univariate ACE decomposition model for school readiness. Then, to estimate if the contributions of A, C and E to school readiness varied as a function of child-care attendance, we conducted a basic continuous GxE interaction analysis [27]. This model allows to test whether child-care attendance has a moderating effect on any or all of its A, C, and E components, while controlling for its main phenotypic effect on school readiness (as in Fig. 1). In a basic continuous GxE interaction analysis, the variance of the trait is partitioned into latent A, C and E components, which are all expressed as a linear function of the moderator (here, child-care attendance). To do so, the GxE analysis simultaneously estimates 8 parameters: ${\beta }_{m}$ the regression phenotypic coefficient (i.e., the regression coefficient estimate of the effect of child-care on school readiness); A, C, E, which corresponds to the main ACE estimates for the outcome variable (i.e., school readiness); ${\beta }_{a}$, ${\beta }_{c}$ and ${\beta }_{e}$ which represents the effect of the moderator (i.e., child-care attendance) on the ACE parameters for school readiness. Put differently, ${\beta }_{a}$, ${\beta }_{c}$ and ${\beta }_{e}$ corresponds to the linear slope of the ACE parameters (of school readiness) varying as a function of the moderator (child-care attendance).

For each twin pair conditional on the twins’ measured environment moderator (M), the expected trait (school readiness) mean for twin *i* is given by ${M}_{i}=\mu +{\beta }_{m}$ where $\mu$ is the mean of the trait (school readiness). The residual trait variance unexplained by the main effect is then partitioned into A, C and E components as so:

$$Var({T}_{i})=(a+{\beta }_{a}{M}_{i}{)}^{2}+(c+{\beta }_{c}{M}_{i}{)}^{2}+(e+{\beta }_{e}{M}_{i}{)}^{2}$$

And the expected heritability of school readiness for a given child-care attendance would be expressed by

$${A}^{2}=(a+{\beta }_{a}M{)}^{2}$$

We computed two basic continuous GxE interaction analyses. First, we estimated the moderating effect of child-care intensity on school readiness. Second, we estimated the moderating effect of formal child-care age of onset on school readiness. For each of these, the full model with all 8 parameters was estimated. Formal child-care attendance was regressed on the selection factors within the model to account for social selection. Statistical significance was determined at 95% confidence intervals.

## Results

Table 1 presents the means and standard deviations for formal child-care attendance indicators and school readiness as a function of zygosity and sex. There were no zygosity or sex differences in school readiness or child-care attendance indicators. Therefore, for all subsequent analyses, we considered scores for males and females together.

**Table 1:** Standard deviations and analysis of variance by sex and zygosity for school readiness and formal daycare attendance

Variables	All		MZ		DZ		Female		Male		ANOVA p-value		
	*M*	*SD*	*M*	*SD*	*M*	*SD*	*M*	*SD*	*M*	*SD*	z*yg*	*sex*	z*yg* ∗ *sex*
School readiness	42.8	13.0	42.5	12.6	43.0	13.3	43.5	13.1	42.1	12.9	.50	.20	.76
Intensity	6.8	9.4	7.0	9.5	6.6	9.3	6.7	9.5	6.9	9.2	.80	.71	.42
Age of onset	48.1	18.4	48.6	18.9	47.8	18.1	48.0	19.1	48.3	17.7	.24	.33	.28

*MZ* Monozygotic, *DZ* dizygotic, *ANOVA* analysis of variance; zyg/sex/zygsex = p-value associ- ated variance attributable to zygosity/sex/the *xygosity* ∗ *sex* interaction

Table 2 shows the Pearson’s correlations between the child-care variables (intensity and age of onset) and the selection factors. Child-care intensity and child-care age of onset correlate very strongly and negatively (− 0.85***), indeed starting early allowed to be exposed to child-care a greater amount of time. Enrolling in child-care early and for a high number of hours per week was positively associated with school readiness.

**Table 2:** Correlations between control variables, school readiness and daycare intensity and age of onset

	Daycare intensity	Daycare age of onset	School readiness	N
Family income	.27***	− .15**	.30***	1288
Siblings	− .20***	.22***	− .11**	1282
Marital status (intact)	.01	.03	.10*	1277
Health at birth	.02	.04	.10*	1242
Ethnicity (Whites)	− .20***	.16***	.03	1252
Pregnancy smoking	− .12**	.11**	− .18***	1290
Breast feeding (yes)	.15**	− .13**	.16***	1279
Birth weight	.04	− .06¨	.07¨	1204
Maternal age at birth	.09*	.02	.21***	1294
Age at first cycle	− .02	.00	− .02	1292
Sex (male)	.02	.01	− .02	1296
Family functionning	− .07¨	.03	− .07¨	1210
Paternal education	.27***	− .25***	.29***	1168
Maternal education	.20***	− .17***	.30***	1258
Daycare intensity	–	− .85***	.21***	770
Daycare age of onset	− .85***	–	− .14**	843
School readiness	.21***	− .14**	–	908

***p < .001., **p < .01., *p < .05., ¨*p* < .2

For subsequent analyses we selected a more parsimonious covariate set to keep only covariates with significant predictive power. We performed a multivariate hierarchical linear model with the 14 selection factors as independent variable and child-care intensity and age of onset as dependent variables (Supplementary Tables 1–2). We used a hierarchical model to correct for the intrinsic nested structure of twin data (two twins per family). We then performed a stepwise regression analysis, first dropping non-significant covariates then dropping covariates with negligible impacts on the ${r}^{2}$ on the child-care moderators [38]. Four predictive control variables were retained out of this process (Family income, number of siblings, ethnicity and paternal education). Variance inflation factors (VIF), which quantify the degree of multicollinearity between the predictors, are presented in Supplementary Table 3. Covariates with a VIF over five were considered multicollinear. No multicollinearity was found in the covariate set.

Our univariate genetic analysis indicated that school readiness included a large contribution from shared environmental factors (${C}^{2}=.46$, 95% CI [$.30$, $.62$]), and to a lesser extent, from genetic factors (${A}^{2}=.35$, 95% CI [$.21$, $.50$]), as well as unique environmental factors (${E}^{2}=.19$, 95% CI [$.16$, $.24$]).

Table 3 displays the moderation model estimates for both child-care attendance indicators. Child-care attendance intensity, but not age of onset, significantly moderated the shared environment contribution to school readiness. The ${\beta }_{c}$ row represents the effect of the moderator (i.e., child-care attendance) on the ACE parameters for school readiness. For intensity, each additional hour per week of formal child-care was associated with a lower C contribution to school readiness by − 0.17 95% CI $[-.29,-.05]$. For age of onset, each additional month of age of onset increases the C contribution to school readiness by 0.02 95% CI $[-.04,.14]$, but the value did nor reach statistical significance. This moderation was specific to shared environment as child-care quantity indicators did not moderate the additive genetic or unique environment contributions to school readiness.

**Table 3:** Unstandardized parameter estimates with 95% confidence intervals for the full GxE model for daycare intensity and age of onset

	Intensity			Age of onset		
	Est	95% CI	*p*	Est	95% CI	*p*
Additive genetic (A)	− 7.54	[− 9.28, − 5.43]	.00	10.43	[6.60, 13.04]	.00
Shared environment (C)	8.86	[6.87, 10.43]	.00	5.62	[.38, 10.12]	.06
Unique environment (E)	5.38	[4.50, 6.20]	.00	3.98	[2.70, 5.32]	.00
A x daycare moderation (β_*a*_)	− .06	[− .14, .05]	.34	− .05	[− .11, .03]	.23
C x daycare moderation (β_*c*_)	− .17	[− .29, − .05]	.02	.05	[− .04, .14]	.38
E x daycare moderation (β_*e*_)	− .02	[− .07, .04]	.48	.02	[− .01, .05]	.19
Mean	4.63	[− .36, 9.91]	.14	48.81	[39.80, 58.09]	.00
Linear mean moderation	.25	[− .06, .55]	.18	− .10	[− .15, − .04]	.00

*Est* Estimates, *95% CI* 95% confidence intervals

Table 4 displays the fit statistics for the full saturated model and nested sub models for both child-care intensity and age of onset. The least significant parameters were dropped until no parameters could be removed without significant loss of fit. For child-care intensity, the best fitting model, as indicated by AIC, was one with the genetic and non-shared environment moderation were constrained to zero, but not the shared environment moderation. Comparing this model with a model in which all three moderations were constrained to zero led to marginally significant reduction of fit $\chi$ ^2^(1) = 2.908, p = 0.088, suggesting a moderation effect, but limited power. For age of onset, the best fitting model was one with no moderation of either genetic or environmental components.

**Table 4:** Fit statistics for full child-care moderation and nested sub-models

Model	Child-care	EP	AIC	Δ − *2ll*	Δ*df*	p-value
Fully saturated model	Intensity	9	11,716.61	–	–	–
**Drop A and E × child-care intensity moderation**		**7**	**11,713.41**	**0.8**	**2**	**0.67**
Drop A, C and E × child-care intensity moderation		6	11,714.32	3.7	3	0.3
AE model (no C, no moderation)		5	11,727.92	15.3	4	< 0.001
Fully saturated model	Age of onset	9	13,328.53	–	–	–
Drop A and E × child-care age of onset moderation		7	13,327.33	2.8	2	0.25
**Drop A, C and E × child-care age of onset moderation**		**6**	**13,325.38**	**2.85**	**3**	**0.42**
AE model (no C, no moderation)		5	13,341.17	16.65	2	< 0.001

*EP* Estimated parameters, *AIC* Akaike information criterion of the model; Δ − *2ll* difference in − 2log likelihood with the full saturated model; Δ*df* = difference in degrees of freedom with the full saturated model. All comparisons are with respect to the full saturated model. Smaller p-values indicate worse fit. The best fitting models are in bold

**Fig. 2:**
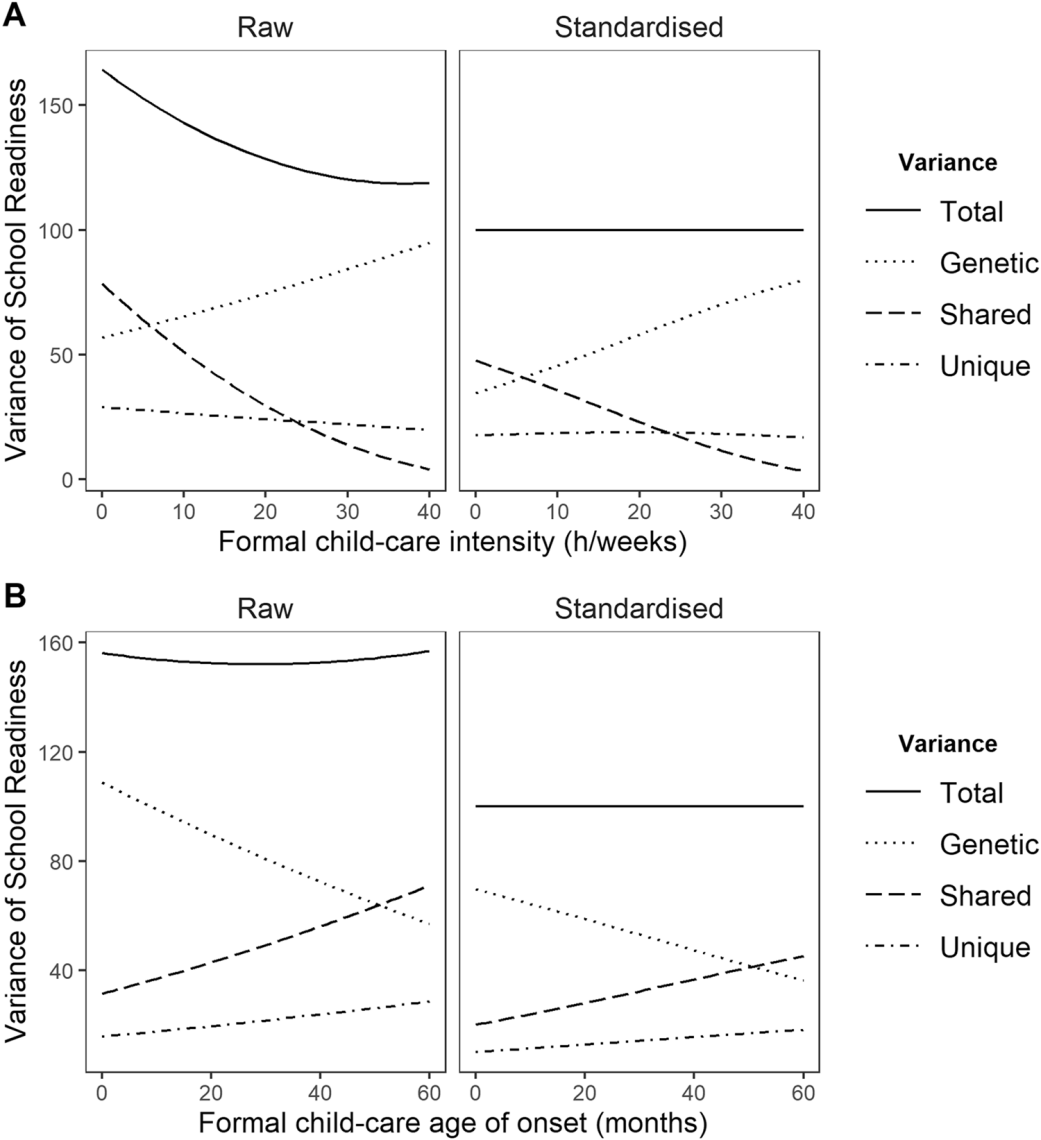
Moderation effect of formal daycare intensity on the genetic, shared environment and unique environment contributions to school readiness. *Note* This figure demonstrates the genetic and environmental variance components for school readiness as a function of child-care attendance. Only the shared environment slope for intensity is significant

Figure 2 presents a graphical summary of the moderation analyses. It displays the moderating effect of formal child-care attendance (the x-axis) on the ACE contribution of school readiness (the y-axis), while taking into account selection factors. On Fig. 2A, child-care intensity was significantly associated with a decrease of C contribution to school readiness (the dashed line), but not the A and E contributions (the dotted and dotted-dashed lines, respectively). Indeed, the slope of A and E do not significantly differ from zero. The right panel displays the same model, but by holding the total variance of school readiness equal to one hundred. This representation allows us to visualize that the relative contribution of A and E increases while the relative contribution of C decreases with increased child-care intensity. Figure 2B depicts the same representation, but for age of onset. No slope was significantly different from zero.

## Discussion

The goal of the present study was to test whether longitudinal child-care attendance, as measured by intensity and age of onset, moderated the genetic and environmental contributions to individual differences in school readiness. The main novel finding was that child-care intensity, that is the average number of hours per week a child spent in child-care between ages 6 and 50 months, was associated with a lower shared environment contribution to school readiness. Shared environment explained 48% of individual differences in school readiness for children not attending formal daycare, but decreased gradually to a mere 3% for children attending formal daycare full time, e.g., 40 h per week. However, the result for age of onset of child-care attendance was not conclusive.

### Formal Child-Care as a Normalizing Environment for Vulnerable Children

The present results are consistent with the view that child-care offsets between-family differences in school readiness, some of these perhaps associated with less stimulating environments. Previous research using a singleton design showed that child-care could compensate, at least partially, for a disadvantaged home life. Indeed, child-care was found to be beneficial for children living in a precarious or low-stimulating household but it had little or no impact on children adequately stimulated at home [6]. For example, children from poorly educated mothers showed a consistent pattern of lower scores on receptive vocabulary, academic readiness, and achievement tests at 6, 7 and 12 years of age in comparison with children of highly educated mothers, but not if they receive formal child-care [4, 17, 18]. This could reflect inequalities between parents' resources to adequately stimulate their children [39]. However, children attending child-care also receive stimulation from child-care providers. As formal child-care centers in this population provide a uniform educational program, child-care attendance intensity could make up for the less optimal learning experiences provided by the family environment.

These phenotypic studies only demonstrated that child-care attenuates the impact of specific family characteristics on children’s preschool cognitive development (e.g., SES, maternal stimulation, maternal education) [4, 6, 17, 18]. By contrast, building on the twin model, the present study showed that child-care attendance over the full preschool period is not only associated with school readiness at 5 years of age but that it decreased more generally the total family-level influence to school readiness (the shared environment contribution). The likely process at play is that child-care attendance acts as a normalizing environment decreasing differences between families [40]. Indeed, even though twins in the same family are virtually identical in their child-care exposure (i.e., they attend the same child-care for the same amount of time per week), and different families differ in their child-care exposure (i.e., children attend different child-care centers), individual differences in school readiness decreases with between family differences in child-care intensity. As formal child-care settings provide uniform educational programs in this population, exposure to cognitive stimulation is likely to vary less in child-care settings than it does between families. This is relevant for child-care policy makers because it poses child-care as a way to ensure every child has an equal access to learning opportunities before school entry. However, restricted access to quality child-care is likely to create a deleterious situation in which children from lower SES families are less likely to enroll in child-care, when they are the ones who appear to benefit the most from it [18, 41].

Tucker-Drob et al. (2012), in their study of the moderating effect of child-care attendance come to very similar conclusions. The current study replicates their findings but goes beyond by showing that a continuous measure aggregating four different time points of formal child-care intensity exerts a similar moderating effect even when controlling for several selection factors into child-care. From a causal inference standpoint, these results could point to a true causal moderating effect. Indeed, our results point to a dose–response relationship between child-care and school readiness. Moreover, our more nuanced finding highlights the importance of intensity of care rather than age of onset as a main driver of the moderation.

### Etiological Contributions to Early Cognitive Development

The present study also provided insights into the environmental and genetic contributions to cognitive abilities in early development. It is well established that the shared environmental contribution to cognitive abilities is high during the preschool years (~ 60%) and gradually decreases from school entry into adolescence, a point at which it becomes negligible [26, 42]. One hypothesis to explain this trend is the enrollment into the school system [43]. In school, children are exposed to a homogeneous learning environment, therefore diminishing environmental variance and differences between families [5, 44]. Although appealing, this hypothesis lacks empirical support mainly because school is mandatory, making it nearly impossible to have variability in school exposure to elucidate whether the observed trend is a normal developmental process or a school effect.

Our results, which build on the natural occurrence of children attending formal child-care settings bypass this limit. Indeed, formal child-care resembles school in its setting and educative purpose. However, it is non-mandatory, so children may attend it or not, at various ages, and with various intensities. This natural heterogeneity in child-care exposure allowed us to observe that formal child-care attendance intensity decreases the contribution of shared environment to cognitive abilities, even when children are enrolled at a very early age. It is therefore probable that child-care creates the same pattern we observe after school entry and that it is these environmental settings and not merely development that causes a decrease in the contribution of shared environment to cognitive abilities.

More importantly, the observed moderation of the intensity of formal child-care attendance suggests that etiological patterns vary within subgroups of a given population. This means that additive etiological models may in fact be misleading. Indeed, results show that under conditions of high intensity, shared environmental contributions to school readiness may actually be negligible at this age.

### Limits of the Present Study

The novelty of our results should be considered within the limits of the study design. First, twins possibly differ from a singleton sample in child-care attendance as with studies of families with numerous children. Indeed, twins from our sample attended child-care with a weekly average of 6.78 h (SD = 9.36; range = 0 to 38.75) which is lower than a same age singleton sample at the same time 14.33 h (SD = 15.06; range = 0 to 46) [18]. Parents with more than one infant may choose to delay child-care entry because of the increased cost. However, results may be generalizable because differences in means are less likely to affect the test for moderation within a sample.

Second, the present study remains observational in nature and child-care conditions were not randomly assigned, but rather selected by the parents making it impossible to infer any causality. For example, it is possible that parents choosing to send their children to child-care are more similar than parents who do not, in which case the observed moderating effect could partially stem from the parents’ choice to use formal child-care. To partial out this confounding effect, we followed sensible recommendations and controlled for several theoretically and empirically relevant factors to reduce the effect of social selection bias [17, 45]. Correcting for these measured confounders gives us more confidence that the moderation could represent a true causal effect. However, other unmeasured confounding factors could be at play. Therefore, further studies should assess the outcome before and after child-care enrollment to assert plausible causality inferences.

Third, intensity has a skewed or L shape distribution, with nearly half of the children scoring zero, because they never went to daycare. It has been shown that non-normal traits can have effects on the results of interaction analyses [27]. However, we are not aware of any studies assessing how distributional properties of the moderator could affect GxE results. The inclusion of a moderators that is not normally distributed is unlikely to have substantially affected the results since the binarization of the moderator yielded similar results.

Lastly, we restricted our analysis to a subtype of child-care, formal child-care, because of its documented higher and more uniform quality, its widespread usage in Quebec (70% of children attending child-care attend formal child-care) [16] and its stronger association with school readiness and school abilities [18]. The fact that the study is rather targeted with regard to childcare settings probably restricts the variance of quality and therefore constitutes a conservative test of the role of these variables. Nonetheless variability in quality remains among formal child-care centers, upholding the possibility that the interaction we found is driven by the variance in child-care quality. Typically, quality is measured directly with specific characteristics such as the caregivers’ training, child-staff ratio, and child-staff relationship. As child-care quality was not directly assessed in this study, we cannot exclude its possible implication in the moderation process whereby parents may be more likely to send their children in higher quality centers. Our results may not be generalizable to other type of child-care of lesser quality.

### Conclusion

In sum, the results reported here support the idea that formal child-care acts as a normalizing environment, possibly buffering the liability stemming from less stimulating home environments during the preschool years. Also, child-care attendance could precipitate the documented decrease of the contribution of shared environment to cognitive abilities after school entry. Further genetically sensitive designs investigating child-care moderating effects should include outcome measures before child-care entry to assert plausible causality inference as well as later outcomes measures to assess if the potential effects remain throughout development. In any case, direct measures of child-care quality are needed to determine if the interaction is driven by a subset of high-quality child-care centers. These studies need to be well powered enough to detect an interaction effect.

### Summary

Daycare attendance improves school readiness and school achievement, especially for children from disadvantaged socio-economic households. More broadly, daycare could potentially decrease the total family contribution to school readiness. In this study, we used a prospective, population-based, and genetically descriptive sample of 648 pairs of twins to explore gene-environment interaction. We assessed how daycare attendance moderates genetic, shared environmental and unshared environmental contributions to school readiness.

We showed that a high number of hours per week decreases the shared environmental contributions to school readiness (but not the genetic and unique environmental contributions). Shared environment explains 48% of individual differences in school readiness for children not attending formal child-care and decreased gradually to a mere 3% for children attending formal child-care full time, e.g., 40 h per week. Age of onset exerted no moderation effect.

Past research has shown that formal daycare reduces the impact of negative specific family characteristics (e.g., low socio-economic status, low maternal stimulation, low maternal education). Our results support the idea that daycare attendance decreases more generally the family-wide influence on school abilities. Daycare could potentially act as a normalizing environment, decreasing differences between families and possibly buffering from the negative effect of less stimulating home environments. These results pose daycare as a mean to ensure every child has an equal opportunity of learning before school entry.

### Supplementary Information

Below is the link to the electronic supplementary material.

Supplementary file1 (DOCX 35 kb)
