# Supplementary material for: The Intensity of Formal Child-Care Attendance Decreases the Shared Environment Contribution to School Readiness: A Twin Study
Source: Child Psychiatry Hum Dev. 2022 Oct 21;55(4):882–92. doi: 10.1007/s10578-022-01440-6 (PMC11245436; doi:10.1007/s10578-022-01440-6)
Supplement: Supplementary file 1 — Supplementary file1 (DOCX 35 kb) [file 10578_2022_1440_MOESM1_ESM.docx]

**Supplementary Material**

**Table 1**

*Child-care intensity: selection of covariates with stepwise hierarchical linear regression*

|  | Full covariates | | |  | Significant covariates | | |  | Remaining covariates | | |  |
| --- | --- | --- | --- | --- | --- | --- | --- | --- | --- | --- | --- | --- |
|  | 𝑟^2^ = 0.221 | | |  | 𝑟^2^ = 0.212 | | |  | 𝑟^2^ = 0.195 | | |  |
| Variables | Estimate | SE | P | Estimate | | SE | P | Estimate | | SE | P | |
| Family income | 0.299 | 0.071 | 0.000 | 0.315 | | 0.071 | 0 | 0.256 | | 0.069 | 0 | |
| Number of siblings | -0.191 | 0.047 | 0.000 | -0.178 | | 0.043 | 0 | -0.187 | | 0.043 | 0 | |
| Ethnicity (White) | -0.301 | 0.070 | 0.000 | -0.977 | | 0.229 | 0 | -0.902 | | 0.229 | 0 | |
| Paternal education | 0.135 | 0.062 | 0.030 | 0.128 | | 0.06 | 0.032 | 0.135 | | 0.061 | 0.027 | |
| Marital status (intact) | 0.144 | 0.067 | 0.031 | 0.146 | | 0.069 | 0.034 |  | |  |  | |
| Breast feeding (Yes) | 0.052 | 0.049 | 0.292 |  | |  |  |  | |  |  | |
| Birth weight | 0.038 | 0.059 | 0.517 |  | |  |  |  | |  |  | |
| Maternal education | -0.012 | 0.062 | 0.846 |  | |  |  |  | |  |  | |
| Pregnancy smoking | 0.014 | 0.033 | 0.684 |  | |  |  |  | |  |  | |
| Age at first cycle | 0.000 | 0.062 | 0.995 |  | |  |  |  | |  |  | |
| Health at birth | -0.039 | 0.039 | 0.313 |  | |  |  |  | |  |  | |
| Sex (Male) | -0.014 | 0.038 | 0.719 |  | |  |  |  | |  |  | |
| Maternal age at birth | 0.017 | 0.054 | 0.748 |  | |  |  |  | |  |  | |
| Family functioning | -0.022 | 0.053 | 0.670 |  | |  |  |  | |  |  | |

*Note.* The most parsimonious selection of covariates was chosen

**Table 2**

*Child-care age of onset: selection of covariates with stepwise hierarchical linear regression*

|  | Full covariates | | |  | Significant covariates | | |  | Remaining covariates | | |  |
| --- | --- | --- | --- | --- | --- | --- | --- | --- | --- | --- | --- | --- |
|  | 𝑟^2^ = 0.174 | | |  | 𝑟^2^ = 0.160 | | |  | 𝑟^2^ = 0.145 | | |  |
| Variables | Estimate | SE | P | Estimate | | SE | P | Estimate | | SE | P | |
| Family income | -0.154 | 0.075 | 0.041 | -0.114 | | 0.068 | 0.094 | -0.109 | | 0.068 | 0.108 | |
| Number of siblings | 0.234 | 0.049 | 0.000 | 0.251 | | 0.046 | 0 | 0.228 | | 0.044 | 0 | |
| Ethnicity (White) | 0.674 | 0.175 | 0.000 | 0.641 | | 0.182 | 0 | 0.662 | | 0.188 | 0 | |
| Paternal education | -0.166 | 0.059 | 0.005 | -0.16 | | 0.058 | 0.006 | -0.166 | | 0.058 | 0.004 | |
| Birth weight | -0.124 | 0.059 | 0.034 | -0.093 | | 0.047 | 0.048 |  | |  |  | |
| Health at birth | 0.085 | 0.041 | 0.039 | 0.088 | | 0.041 | 0.033 |  | |  |  | |
| Breast feeding (Yes) | -0.060 | 0.101 | 0.553 |  | |  |  |  | |  |  | |
| Marital status (intact) | -0.492 | 0.305 | 0.107 |  | |  |  |  | |  |  | |
| Maternal education | -0.016 | 0.059 | 0.786 |  | |  |  |  | |  |  | |
| Pregnancy smoking | -0.003 | 0.036 | 0.942 |  | |  |  |  | |  |  | |
| Age at first cycle | -0.065 | 0.061 | 0.285 |  | |  |  |  | |  |  | |
| Sex (Male) | -0.034 | 0.075 | 0.648 |  | |  |  |  | |  |  | |
| Maternal age at birth | 0.043 | 0.056 | 0.442 |  | |  |  |  | |  |  | |
| Family functioning | 0.008 | 0.049 | 0.877 |  | |  |  |  | |  |  | |

*Note.* The most parsimonious selection of covariates was chosen

**Table 3**

*Variance inflation factor (VIF) for all covariates*

| Variables | Age of onset | Intensity |
| --- | --- | --- |
| Family income | 1.688 | 1.741 |
| Number of siblings | 1.222 | 1.225 |
| Ethnicity (White) | 1.203 | 1.175 |
| Paternal education | 1.494 | 1.175 |
| Marital status (intact) | 1.018 | 1.123 |
| Breast feeding (Yes) | 1.234 | 1.230 |
| Birth weight | 1.614 | 1.720 |
| Maternal education | 1.583 | 1.599 |
| Pregnancy smoking | 1.226 | 1.299 |
| Age at first cycle | 1.488 | 1.580 |
| Health at birth | 1.091 | 1.100 |
| Sex (Male) | 1.038 | 1.044 |
| Maternal age at birth | 1.257 | 1.267 |
| Family functioning | 1.093 | 1.095 |
